# Alternative feed ingredients in the finisher diets for sustainable broiler production

**DOI:** 10.1038/s41598-020-74950-9

**Published:** 2020-10-20

**Authors:** Ahmed A. El-Deek, Ahmed A. A. Abdel-Wareth, Mona Osman, Mohammed El-Shafey, Ayman M. Khalifah, Alaa E. Elkomy, Jayant Lohakare

**Affiliations:** 1 Poultry Production Department, Alexandria University, Alexandria, 21545 Egypt; 2 Department of Animal and Poultry Production, Faculty of Agriculture, South Valley University, Qena, 83523 Egypt; 3 Ismailia/Misr for Poultry Production, 28, Ismailia, Egypt; 4 Livestock Department, Arid Land Cultivating Research Institute, City of Scientific Research and Technology Applications (SRTA City), 21934, New Borg El Arab, Egypt; 5 Faculty of Desert and Environmental Agriculture, Matrouh University, Matrouh, Egypt; 6 Department of Agriculture-Animal Science Option, University of Arkansas At Pine Bluff, Pine Bluff, AR 71601 USA

**Keywords:** Biological techniques, Developmental biology, Microbiology, Physiology, Systems biology, Zoology, Environmental sciences

## Abstract

The main objective of this study was to evaluate the utilization of alternative protein feed ingredients including sunflower meal (SFM), corn gluten meal (CGM), and dried distillers’ grains with solubles (DDGS) as a mixture in a partial replacement of soybean meal (SBM) in broiler finisher diets with different protein levels and also to evaluate their effect on birds’ performance, environmental aspects of litter, cecal microbes, and economic prospects. A total of 576 (19 days old) Cobb 500 broiler chicks were fed eight finisher diets consisting of 4 control (CTL) diets based on SBM with different crude protein (CP) levels (CTL21, CTL20, CTL19, and CTL18, containing 21%, 20%, 19%, and 18% CP, respectively) and 4 test diets with alternative protein sources (APS21, APS20, APS19, and APS18, containing 21%, 20%, 19%, and 18% CP, respectively) using a 15% combination of alternative protein sources (2.5% CGM, 5% SFM, and 7.5% DDGS) until 35 days of age. The results indicated that birds fed test diets APS21 and APS20 recorded the highest (*P* < 0.05) body weight compared to other treatments, but it was not different than the CTL diets fed at these CP levels. The birds fed CTL18 or APS18 recorded the worst feed conversion ratio (FCR) compared to other treatments. Moreover, birds fed test diet containing APS21 recorded better (*P* < 0.05) European performance efficiency factor and better economic efficiency when compared to other treatments, but it was not different than CTL21. In addition, birds fed diets APS21 and CTL19 showed significantly increased litter *Lactobacillus* spp. (*P* < 0.05) compared to other treatments. Cecal *Lactobacillus* spp. and *Escherichia coli* (*E*. *coli*) were not affected by CTL or APS diets. The counts of cecal *Salmonella* spp*.* increased in the CTL21 group compared to other groups. In conclusion, alternative feed ingredients (protein sources) in broiler finisher diets have positive effects in a sustainable way on the productive performance, litter and cecal microbial counts, and improved economic efficiency when compared to CTL diets.

## Introduction

The globalization of the food value chain is increasing rapidly, and it is necessary to address the challenges associated with it in a sustainable way. To overcome the challenges, the poultry sector will have to focus more on the sustainability of production and cheap protein sources. The animal production efficiency could be improved by reducing the output of nutrients as waste to the environment^1^. The production of broiler chickens must achieve the objective of sustainability, as climate change concerns have major effects on its future growth performance^2^.

Feed cost represents approximately 65–75% and is considered the major cost of poultry production^3^. Many attempts have been made to decrease the cost of feeding to the minimum levels. These attempts include replacing the expensive feedstuffs by cheaper and more abundant by-products to support the sustainability of poultry production^4^. Soybean meal (SBM) is often the major dietary plant protein source in broiler diets, and other protein sources other than SBM are used occasionally at competitive prices^4,5^. However, there is a range of possible alternative feed ingredients that can partially or fully replace SBM in poultry diets^6^.

From a nutritional point of view, the current nutritional strategy is to meet the nutrient requirements of broiler chickens and to improve the feed efficiency, which may decrease the nutrient excreted in manure^1^. Poultry farmers are typically reducing the protein and increasing the energy contents throughout the finisher period of broiler chickens^7^. The cost of feed generally declines as the protein content is reduced, and the optimum time for changing diet densities is of economic importance. Due to feeding and genetic improvements, the time required to reach market weights has been reduced, leading to shorter durations of feeding various diets^8^.

The plant-based protein sources used for broilers in the poultry industry, especially SBM, had a shortage since 2005^9^, so other protein sources are expected to be included at high inclusion levels for optimum production. Several studies have been conducted using different plant protein sources to replace the SBM in animal feed such as canola meal (CNM), sunflower meal (SFM), rapeseed meal (RSM), cottonseed meal (CSM), corn gluten meal (CGM), and dried distillers’ grains with solubles (DDGS)^10,11^. DDGS and SFM are new feed ingredients and may be used as an alternative source of protein in animal and poultry diets. Thus, coming up with alternative protein sources such as DDGS, CGM, and SFM that are cheap and locally available could improve broiler production and at the same time improve the economic status of the poultry industry. Therefore, the aim of this study was to formulate sustainable finisher diets containing a mixture of DDGS, CGM, and SFM as a partial alternative protein source (APS) for SBM at different crude protein (CP) levels (21%, 20%, 19%, and 18% CP, respectively) and to study their effect on the growth performance, litter content, and economic efficiency of Cobb 500 broiler chicks. We tested these CP levels in the finisher diets of broilers because the finisher diets in commercial broiler formulations worldwide use these CP levels in the diet.

## Materials and methods

The experiment was conducted at Ismailia/Misr Company for poultry production, Sarapium district, Ismailia, Egypt. The laboratory analyses of this study were done at the Poultry Research Center, Faculty of Agriculture, Alexandria University, and the laboratory of Livestock Research Department, Arid Land Cultivating Research Institute, City of Scientific Research and Technology Applications, New Borg El Arab, Egypt.

### Experimental design and diets

A total of 576 unsexed 1-day-old Cobb 500 chicks were procured from a commercial hatchery of Ismailia/Misr Company. All chicks were fed a starter diet with 23% crude protein (CP) from 1 to 18 days of age. From the 19th day, birds were fed finisher diets until 35 days of age. Broiler chicks were distributed into 24 floor pens (8 treatments × 3 pens per treatment × 24 chicks per pen). The eight treatments consist of 4 control (CTL) diets (CTL21, CTL20, CTL19, and CTL18, containing 21%, 20%, 19%, and 18% CP, respectively) and 4 test diets with alternative protein sources (APS21, APS20, APS19, and APS18, containing 21%, 20%, 19%, and 18% CP, respectively).

The test diets of the finisher phase were based on a combination of APS (2.5% corn gluten meal, 5% sunflower meal, and 7.5% dried distillers’ grains with solubles (DDGS)) as a fixed entity (15%) for all levels of protein studied. The percentage of inclusion of alternative ingredients to replace SBM was selected to formulate diets to keep the replacement level of APS constant across diets of different CP levels. Experimental diets were formulated to contain 3100 kcal of ME/kg for starter and finisher diets in either CTL or APS test groups. All diets contained additives such as phytase, coccidiostats, and multienzymes. The multienzyme called COMBO Enzyme Blend consists of the following: cellulase, 75,000 CU units/kg; fungal amylase, 30,000 SKB units/kg; fungal protease, 1,000,000 HUT units/kg; neutral protease, 100,000 PC units/kg; alkaline protease, 1.2 Anson units/kg; xylanase, 20,000 × U units/kg; beta-glucanase, 20,000 BG units/kg; hemicellulase, 20,000 HCU units/kg; and lipase, 75,000 FIP units/kg. Ingredients and calculated analysis of broiler diets used in the experiment are shown in Table 1.

**Table 1:** Ingredients and calculated analysis of finisher diets.

Ingredients (%)	Finisher diets							
	CTL 21% CP	APS 21% CP	CTL 20% CP	APS 20% CP	CTL 19% CP	APS 19% CP	CTL 18% CP	APS 18% CP
Yellow corn	60.88	55.879	63.88	58.73	67.18	61.58	70.17	64.18
SBM (48% CP)	33.30	22.60	30.80	20.00	28.00	17.35	25.46	15.00
Gluten meal (62% CP)	–	2.50	–	2.50	–	2.50	–	2.50
DDGS (31% CP)	–	7.50	–	7.50	–	7.50	–	7.50
SFM (28% CP)	–	5.00	–	5.00	–	5.00	–	5.00
Soy oil	2.90	3.60	2.40	3.25	1.90	2.90	1.50	2.50
Limestone	1.75	1.75	1.75	1.75	1.75	1.75	1.75	1.85
Di-calcium phosphate	0.20	0.200	0.20	0.20	0.15	0.20	0.20	0.20
Common salt	0.45	0.45	0.45	0.45	0.45	0.45	0.45	0.45
Vitamin premix^1^	0.10	0.10	0.10	0.10	0.10	0.10	0.10	0.10
Mineral premix^2^	0.20	0.20	0.20	0.20	0.20	0.20	0.20	0.20
L-lysine HCL (98%)	–	0.050	–	0.15	–	0.25	–	0.30
DL-methionine (98%)	0.15	0.10	0.15	0.10	0.10	0.15	0.10	0.15
Mixed enzymes^3^	0.03	0.03	0.03	0.03	0.03	0.03	0.03	0.03
Phytase^4^	0.005	0.005	0.005	0.005	0.005	0.005	0.005	0.005
Coccidiostat^5^	0.02	0.02	0.02	0.02	0.02	0.02	0.02	0.02
Antimycotoxin^6^	0.01	0.01	0.01	0.01	0.01	0.01	0.01	0.01
Probiotic^7^	0.006	0.006	0.006	0.006	0.006	0.006	0.006	0.006
Total	100	100	100	100	100	100	100	100
**Calculated analysis**								
ME (kcal/kg)	3100	3100	3100	3100	3100	3100	3100	3100
Crude protein (%)	21	21	20	20	19	19	18	18
Fat (%)	2.75	3.4	2.85	3.48	2.94	3.57	3.02	3.65
Fiber (%)	2.45	3.97	2.44	3.95	2.43	3.94	2.41	3.92
Calcium (%)	0.77	0.76	0.77	0.76	0.75	0.75	0.75	0.75
Phosphorus (%)	0.38	0.38	0.40	0.40	0.39	0.42	0.43	0.44
Total lysine (%)	1.05	1.05	1.13	1.20	1.01	1.04	1.08	1.05
Methionine (%)	0.45	0.51	0.48	0.49	0.49	0.50	0.47	0.48
Cysteine (%)	0.28	0.32	0.28	0.30	0.28	0.31	0.28	0.30
Meti + cyst (%)	0.73	0.83	0.76	0.79	0.77	0.81	0.75	0.78
Arginine (%)	1.07	1.00	1.06	1.02	1.03	1.04	1.00	1.01

*CTL* soybean meal only, *APS* alternative protein sources containing soybean meal, *DDGS* sunflower meal, and corn gluten meal.

^1^Vitamin premix provides per kg diet Vit. A, 12,000 IU; Vit. E, 400 mg; Vit. Bl, 2 mg; Vit. B2, 160 mg; Vit. B6, 5 mg; Vit. B12, 0.012 mg; niacin, 45 mg; pantothenic acid, 12 mg; Vit. K, 3 mg; Vit. D3, 3000 IU; biotin, 0.070 mg; and folic acid, 2 mg.

^2^Trace mineral premix provides per kg diet choline, 3600 mg; copper, 10 mg; iodine, 1 mg; iron, 30 mg; manganese, 100 mg; zinc, 600 mg; selenium, 0.4 mg; and cobalt, 0.100 mg.

^3^COMBO Enzyme Blend consists of the following: cellulase, 75,000 CU units/kg; fungal amylase 30,000, SKB units/kg; fungal protease, 1,000,000 HUT units/kg; neutral protease, 100,000 PC units/kg; alkaline protease, 1.2 Anson units/kg; xylanase, 20,000 × U units/kg; beta-glucanase, 20,000 BG units/kg; hemicellulase, 20,000 HCU units/kg; and lipase, 75,000 FIP units/kg.

^4^AXTRA PHY 10,000 TPT, 6-phytase 10,000 FTU/g.

^5^Diclazuril 500 mg, ATOZURIL (ATco pharma).

^6^MYCOFIX Select 3, feed additives that protect broiler health by deactivation of mycotoxins.

^7^ENVIVA Pro 202 GT, *Bacillus subtilis* 2.5E CFU/gm.

The chemical analysis of SBM and APS including CGM, SFM, and DDGS is presented in Table 2.

**Table 2:** Chemical compositions of alternative feed sources.

Feedstuffs	CP (%)	ME (Kcal/kg)	Fat (%)	Fiber (%)	Calcium (%)	Total phosphorus (%)
Corn	8.2	3370	3.9	2.3	0.02	0.26
Soybean meal	44	2240	0.9	6	0.32	0.65
Corn gluten meal	62	3700	2.2	2	0.07	0.48
DDGS	31	2766	11	9	0.07	0.7
Sunflower meal	28	1400	2	25	0.34	1

### Birds, housing, and management

The Institutional Animal Ethics Committee of Alexandria University approved the field experiment, and all the methods were performed in accordance with the guidelines of the Egyptian Research Ethics Committee and the guidelines contained in the Guide for the Care and Use of Laboratory Animals. Broiler chicks were kept on clean and fumigated floor pens in a controlled environmental room under similar management conditions. The pen size was 200 cm × 150 cm × 100 cm (L × B × H), and the wood shaving was used as a litter material. Chicks were provided with 24-h artificial lighting daily during the whole experimental period. The gas heater was used to provide the chicks with the heat needed for brooding. Finisher diets were provided in the form of pellets of 3 mm. An ambient temperature program was maintained at 33 °C from placement until 4 days of age, 32 °C from 5 to 9 days of age, 29 °C from 10 to 14 days of age, 27 °C from 15 to 23 days of age, 25 °C from 24 to 28 days of age, and 23 °C from 28 to 35 days of age. Experimental diets and water were offered for ad libitum consumption throughout the experimental phases. The assembly of each pen included a bell drinker and a tube feeder.

### Determination of productive performances

Data on body weight (BW) and feed intake (FI) were determined at the initial and end of each phase. From these data, body weight gain (BWG) and feed conversion ratio (FCR) were computed. The FCR was determined as the ratio between total FI and the total BWG per bird per replicate. Livability was monitored daily by recording and collecting the number of broilers that died, which were then taken for postmortem examination. Livability percentage was then calculated by using the formula ((total number of live birds after the experiment/Initial total number of birds) × 100). The economic efficiency of the dietary inclusion of alternative protein sources was calculated as the total costs needed to obtain one-kilogram BWG according to Kalia et al^12^. The European performance efficiency factor (EPEF) was calculated at the end of the experimental period. The following equation was applied to obtain the EPEF^13^:

$$\mathrm{EPEF}=\frac{Final \,BW,\mathrm{ kg }\times \mathrm{Livability\%}}{Age, days \times FCR}\times 100.$$

### Determination of *Lactobacillus*, *E. coli*, and *Salmonella/Shigella*

At the end of the experiment, 15 birds from each group (5 birds/pen) were selected representing the pen and were decapitated by cervical dislocation, and exsanguination by severing the jugular vein. The carcasses were subsequently opened, and the entire gut tract was removed aseptically. The gut tract was then divided into sections that were ligated with light twine before being separated. The ceca were collected and sealed in sterile bags filled with 50 mL of ice-cold cryoprotective broth (i.e., pre-reduced sterile brain heart infusion broth containing 20% vol/vol glycerol) suitable to maintain the viability of intestinal bacteria^14^ and were immediately stored at − 80 °C for subsequent analyses. For all analytical procedures, deep-frozen ceca per bird were thawed for 20 min and removed from storage bags. Cecal digesta contents were then aseptically emptied in a new sterile bag and were immediately diluted tenfold (i.e., 10% wt/vol) with sterile ice-cold anoxic PBS (0.1 M, pH 7.0) and subsequently homogenized for 3 min. Digesta slurries were then processed as follows. Each cecal digesta homogenate in PBS (1 mL) was serially diluted from 10^–1^ to 10^–7^. Dilutions were subsequently plated on duplicate selective agar media for the enumeration of target bacterial groups. In particular, *Lactobacillus* spp., *E. coli*, and *Salmonella* spp. were enumerated using MRS agar, MacConkey agar, and *Salmonella* agar, respectively^15^. Plates were then incubated at 39 °C for 24–72 h, and colonies were counted. Results were expressed as base-10 logarithm colony forming units per gram of cecal digesta. The following selective culture media were used: MRS agar (Merck) for *Lactobacillus* spp., RAMBACH (Merck) for the *Salmonella* sp., CHROMOCULT (Merck) for *E. coli*.

### Determination of litter compositions

Litter (wood shaving) was sampled (15 samples) from each treatment at the end of the experiment. Each sample corresponded to subsamples taken from 15 random places in a zigzag pattern, and they were obtained from the full depth of the litter (away from the feeders and drinkers). The random litter subsamples were thoroughly mixed and homogenized, and 250 g was weighed and delivered to the laboratory for further processing. A fraction of each sample was immediately dried at 80 °C for 48 h, while the rest of the sample was ground to pass through a 2 mm sieve and frozen at − 20 °C in airtight containers until further analysis. Litter moisture content was determined as loss in weight after oven drying for 48 h at 65 °C, and pH was measured using deionized water to litter ratio of 5:1 (wt:wt). Total nitrogen was determined according to AOAC^16^. Bacterial enumeration for *Lactobacillus* spp., *E. coli,* and *Salmonella* spp. was done for litter samples with the same method of cecal digesta on freshly collected samples.

### Statistical analysis

The model used was one-way ANOVA to study the effect of different dietary treatments. The statistical model was as follows:

$$Y_{ij} = \mu + T_{i} + e_{ij}$$

where *Y*_*ij*_ is the observed value of the dependent variable, *µ* is the overall mean, *T* is the effect of dietary treatments, and *e*_*ij*_ is the experimental random error.

Analysis of variance of obtained data was computed using the general linear model (GLM) and one-way ANOVA procedures according to SPSS^17^. Significant differences among means were evaluated using Duncan’s multiple range test^18^ when significant *P* values were obtained. Pen was the experimental unit for all analyses.

## Results

### Production performance

The performance results showed that birds fed CTL21 diet recorded the highest (*P* < 0.05) BW (1809.22 g), and this value did not differ significantly from the values of birds fed test diet APS21, CTL20 and test diet APS20 since they recorded 1763.48 g, 1765.12 g, and 1748.35 g, of BW respectively (Table 3). On the other hand, groups fed CTL21 and CTL20 diets had a higher (*P* < 0.01) BW compared with birds fed CTL19, APS19, CTL18, and APS18 since they recorded 1568.47 g, 1678.25 g, 1520.78 g, and 1544.84 g BW, respectively, but the group supplied with test diet APS19 was statistically equal to the group fed test diet APS20. Likewise, the results showed that birds fed CTL21 and CTL20 diets recorded the significant highest BWG (1260 g and 1237 g, respectively) during the finisher phase (19–35 days), and these values did not differ from the values of the birds fed test diets APS21 and APS20, since they recorded 1216 g and 1220 g, respectively. On the other hand, groups fed CTL21 and CTL20 diets had a higher BWG (*P* < 0.01) compared with birds in groups APS19, CTL19, APS18, and CTL18 as they recorded 1142 g, 1038 g, 1023 g, and 1002 g, respectively. However, the group supplied with test diet APS19 showed significantly higher BW, BWG, and FI than the CTL19 group.

**Table 3:** Body weight (BW) and body weight gain (BWG) of Cobb broilers fed starter diets for 19 days of age and finisher diets containing alternative protein sources at the levels of 21%, 20%, 19%, and 18% CP for 35 days.

Treatments	BW (g) 19 d	BW (g) 35 d	BWG (g) 19–35 d	Feed intake (g) 19–35 d	FCR 19–35 d
CTL21%	549	1809^a^	1260^a^	2019^b^	1.61^b^
APS21%	547	1763^a^	1216^ab^	2097^a^	1.73^b^
CTL20%	528	1765^a^	1237^a^	2098^a^	1.70^b^
APS20%	528	1748^ab^	1220^ab^	2117^a^	1.75^b^
CTL19%	529	1568^c^	1038^c^	1798^d^	1.76^b^
APS19%	535	1678^b^	1142^b^	1965^c^	1.73^b^
CTL18%	521	1520^c^	1002^c^	2039^b^	2.04^a^
APS18%	517	1544^c^	1023^c^	2093^a^	2.05^a^
SEM	3.255	9.125	25.750	23.615	0.054
Significance test	NS	**	**	**	*

^a–d^Means within columns with no common superscripts are significantly different.

*CTL* soybean meal only, *APS* alternative protein sources containing soybean meal, *DDGS* sunflower meal, and corn gluten meal, *SEM* standard error of means, *NS* not significant.

*P < 0.05; **P < 0.01.

Concerning FI, the results showed that birds in groups APS20, CTL20, APS21, and APS18 recorded the highest (*P* < 0.05) FI (2117 g, 2098 g, 2097 g, and 2093 g, respectively) during the finisher phase. Birds in groups CTL18 and APS18 showed the worst FCR as they recorded 2.05 and 2.04 kg feed consumption/kg gain compared to other groups. However, the opposite was true for birds in groups CTL21, APS21, CTL20, APS20, CTL19, and APS18 as no differences were detected in the FCR (Table 3). Results showed that incorporating 15% combination of SFM, CGM, and DDGS in the diets during finisher phase in APS21 group required less SBM supplementation and could be used for sustainable broiler production.

### Livability, economic efficiency, and European performance efficiency factor

Results of livability were not significantly affected by finisher diets or dietary treatments (Table 4). A highly significant difference (*P* < 0.01) was observed in the EPEF among different groups; birds fed finisher diets CTL21 recorded the highest EPEF (329.87%) which was statistically equal to birds fed test diet APS21 and CTL20 diet (305.86% and 303.48%, respectively) but was different from birds fed APS20, APS19, CTL19, CTL18, and APS18 with EPEF values as 296.04%, 288.50%, 266.91%, 231.62%, and 230.14%, respectively.

**Table 4:** Livability, economic efficiency, and European performance efficiency factor of Cobb broilers fed starter diets and finisher diets containing alternative protein sources at the levels of 21%, 20%, 19%, and 18% of CP.

Treatments	Livability (%)	Economic efficiency	European performance efficiency factor (%)
**Protein level during finisher diets**			
CTL21%	95.89	99.38^a^	329.87^a^
APS21%	96.17	100.00^a^	305.86^ab^
CTL20%	95.58	97.17^a^	303.48^ab^
APS20%	95.40	98.77^a^	296.04^bc^
CTL19%	94.92	98.56^a^	266.91^c^
APS19%	95.85	98.97^a^	288.50^bc^
CTL18%	96.13	87.11^b^	231.62^d^
APS18%	94.49	82.62^b^	230.14^d^
SEM	1.13	4.64	10.5
Significance test	NS	*	**

*CTL* soybean meal only, *APS* alternative protein sources containing soybean meal, *DDGS* sunflower meal, and corn gluten meal, *SEM* standard error of means, *NS* not significant.

*P < 0.05; **P < 0.01.

^a–c^Means within columns with no common superscripts are significantly different for each analysis.

Birds fed test diets with APS and CTL diets at 21, 20, and 19% CP levels showed better economic efficiency than the diets at 18% CP level (Table 5). Birds fed test diet APS21 showed the best economic efficiency compared to other groups.

**Table 5:** Litter analyses of Cobb broilers fed finisher diets containing alternative protein sources at the levels of 21%, 20%, 19%, and 18% of CP.

Treatments	Moisture (%)	pH	Total N (%)	Ash (%)
CTL21%	27.23	7.06	3.47	12.33
APS21%	27.28	7.13	3.45	12.37
CTL20%	27.34	7.00	3.52	12.33
APS20%	27.20	6.97	3.51	12.35
CTL19%	27.30	7.00	3.41	12.27
APS19%	27.30	6.99	3.47	12.26
CTL18%	27.33	7.01	3.43	12.38
APS18%	27.30	6.93	3.44	12.28
SEM	1.29	0.34	0.46	0.43
Significant	NS	NS	NS	NS

*CTL* soybean meal only, *APS* alternative protein sources containing soybean meal, *DDGS* sunflower meal, and corn gluten meal, *NS* not significant, *SEM* standard error of means.

### Environment aspects and litter composition

Mean values of litter content including moisture, pH, total nitrogen, and ash percentage were statistically similar as they were not affected by CTL or APS finisher diets at different protein levels (Table 5). The overall mean of moisture was 27.28%, pH was 7.00, total N was 3.46%, and ash was 12.31%. The count of litter *Lactobacillus* spp. was significantly (*P* < 0.01) influenced by CTL or APS finisher diets (Table 6); results showed that birds fed CTL19 diet recorded the highest value of litter *Lactobacilli* count with a value of 6.24 log CFU/g compared with other groups.

**Table 6:** Litter and cecal bacteria count of Cobb broilers fed starter and finisher diets containing alternative protein sources at the levels of 21%, 20%, 19%, and 18% of CP.

Treatments	Litter bacteria count (log CFU/g)			Caecum bacteria count (log CFU/mL)		
	*Lactobacillus*	*E. coli*	*Salmonella*	*Lactobacillus*	*E. coli*	*Salmonella*
**Protein level and sources during finisher diets**						
CTL21%	5.79^bc^	1.70	0.85^c^	5.64	5.99	6.13^a^
APS21%	6.06^ab^	1.74	2.04^a^	5.77	5.88	3.99^b^
CTL20%	5.53^cd^	1.72	0.00^d^	5.79	5.91	2.06^c^
APS20%	5.85^bc^	1.72	1.05^b^	5.85	5.79	3.48^bc^
CTL19%	6.24^a^	1.66	0.68^c^	5.96	5.91	2.17^c^
APS19%	5.68^bcd^	1.69	1.55^b^	6.08	5.73	2.75^bc^
CTL18%	5.38^d^	1.56	0.00^d^	5.67	5.80	0.00^d^
APS18%	5.69^bcd^	1.57	0.00^d^	5.81	5.76	0.00^d^
SEM	0.295	0.211	0.340	0.183	0.145	1.092
Significance test	**	NS	**	NS	NS	**

*CTL* soybean meal only, *APS* alternative protein sources containing soybean meal, *DDGS* sunflower meal, and corn gluten meal, *NS* not significant, *SEM* standard error of means.

**P < 0.01.

^a–d^Means within columns with no common superscripts are significantly different.

The results of the litter *E. coli* count were not influenced by CTL or APS diets. The overall mean of litter *E. coli* count was 1.68 log CFU/g (Table 6). The counts of litter *Salmonella* spp. were significantly (*P* < 0.01) affected by CTL and APS diets, and this showed that litter *Salmonella* spp. counts increased with APS diets compared to CTL diets at 21%, 20%, and 19% CP levels but it was absent in CTL18 and APS18 groups (Table 6).

### Caecum bacteria count

Cecal *Lactobacillus* spp. and *E. coli* counts were not influenced by CTL and the APS dietary treatments as the overall mean of the cecal *Lactobacillus* spp. count was 5.85 log CFU/mL and the overall mean of the cecal *E. coli* count was 5.85 log CFU/mL (Table 6). The cecal *Salmonella* spp. count increased with CTL diet at the level of 21% CP compared to other groups with the absence of cecal *Salmonella* spp. at the level of 18% CP in CTL or APS diet.

## Discussion

The growth performance data including BW, BWG, FI, and FCR revealed that incorporation of SFM, CGM, and DDGS at 15% level by replacing SBM in the diets containing 21% and 20% CP showed no negative effects on performance and could be used for sustainable broiler production during the finisher period (19–35 days of age). The growth performance was higher in the APS19 group than the CTR19 showing positive effects of APS supplementation. It is apparent from the growth performance data in the current study that sufficient CP was present to support adequate levels of indispensable amino acid synthesis, even in birds fed protein levels at 19% and 18% in the CTR and APS diets. These results agree with Gajana et al.^19^, which showed that birds fed finisher diets at 16 to 35 days resulted in improved body weight of broiler chickens. The growth performance of broilers is significantly increased with decreased levels of crude protein in the finisher period^20^. However, in the current study, the higher level of protein (21% and 20% CP) performed better than the lower levels of protein (19% and 18%) in both control and APS diets. The current study shows that plant protein levels in finisher diets significantly affected the FCR of broilers. FCR of broilers can be significantly affected by the amounts of the dietary protein or energy sources21. On the other hand, increasing the dietary energy levels for broilers has significantly improved FCR^21,22^.

No reports were found regarding the effect of combinations of APS including CGM, DDGS, and SFM in broiler diets but other combinations were done^10,11,23,24^. It was observed that the SBM diet decreased FI of broilers as compared to APS diet at each respective CP level in this study. This may possibly be due to differences in feed texture between CTL and APS diets. The overall mean of livability rate recorded in this study for the Cobb 500 strain was 95.51% which is acceptable according to Cobb recommendations.

Our findings clearly indicate that replacing SBM with a combination of SFM, CGM, and DDGS at 15% level of feeding in broiler finisher diets did not adversely affect production responses and caused an insignificant difference in growth and FCR compared to CTR. Previous studies used 15% DDGS as an APS in broiler diets without negative effects on productive performances which are in agreement with other reports^11,23,24^. Likewise, Damron et al.^10^ found that the substitution of CGM for wheat-based products significantly improves growth performance of broiler chickens. However, the combinations of DDGS and canola may adversely affect the percentage of fines and thus influence performance^11^. Drastic reduction of proteins in nutrition program resulted in a significant decrease of body weight and unfavorable FCR, in line with our results that showed a significant decrease in body weight, and FCR in Cobb 500 strain when birds fed 18% protein in CTL or APS diets and 19% protein in CTL diets.

In the current study, the overall mean of total nitrogen (litter and excreta) of broilers was 3.46%. When broilers are fed diets containing low CP digestibility and unbalanced AA profile, more nitrogen will be excreted in the manure^25,26^. The factor which is important to reduce nitrogen losses from manure is to decrease the amount of CP in the diet.

While formulating finisher diets for broilers, the mixing efficiency of feed ingredients must be taken into considerations to optimize the protein levels and excretion of nitrogen and thereby increase the productive performance of chickens. Since the cost of feed represents more than 75% of poultry production^27^, our study focused more on the production efficiency during the finisher dietary phase by changing feed composition. Birds fed test diet with alternative protein sources showed better economic efficiency and EPEF in the APS21 group compared to other APS groups, and in CTL21 group than other CTL groups. The success in the poultry industry depends on the ability of producers to control broiler feed costs. The results of the current experiment agreed with other studies^19,27,28^. It is a great financial advantage to the poultry producer to cut and replace the starter period with the finisher period^6^. Optimizing protein levels during the finisher feeding phase of broilers improves production performances^29^. However, Tavernari et al.^30^ showed that feeding broiler chickens SBM resulted in the highest economic efficiency compared to the broilers receiving diets containing sunflower meal. It could be concluded that changing the dietary starter phase with sustained finisher diets containing APS at different protein levels during 19–35 days of age improved economic return and EPEF.

DDGS supplementation up to 20%^31^ and SFM supplementation up to 14%^32^ can be used in broiler diets without adverse effects on the performance and economic efficiency. Kim et al.^31^ reported that the availability of DDGS as bioprocessed products, combined with their low cost, has made their application as feed sources for broiler chickens more economical. Feeding broilers combination of SFM, CGM, and DDGS in finisher diets or SBM diets did not alter cecal *Lactobacillus* spp., and *E. coli*, and litter *E. coli* counts*.* On the other hand, the cecal *Salmonella* spp. count increased with CTL diet at the level of 21% CP compared to other groups. The *Salmonella* spp. were not detected in CTR18 and APS18 groups either in litter or cecal contents. High litter *Salmonella* counts were observed in the APS groups than their respective counterparts in the CTL at 21–19% CP levels. Similarly, cecal *Salmonella* load was higher in all finisher dietary CP groups except at 18% CP level and the reasons remained obscure. The risk of *Salmonella* contamination on processed broiler carcasses is reduced when carcasses originate from farms with low detectable levels of *Salmonella*^33^. Śliżewska et al.^34^ reported that the quantitative and qualitative composition of microbiota in the broiler gut tract may change due to the effect of feed composition. However, it remains poorly understood how feed compositions affects development and composition of chicken gut microbiota^31,35^. There are different effects of CTL and APS diet compositions on *Lactobacillus* spp., *E. coli*, and *Salmonella* spp. in litter and ceca of broilers, but the values are within the normal bacteriological range^35–38^. Feeding broilers, a combination of SFM, CGM, and DDGS as APS in finisher diets or SBM in CTL diets improved gut microbiota. The balance of the gut microbiota is an important factor improving digestion, healthy gut, and, therefore, optimum performances.

## Conclusion

Based on the results, the inclusion of the combination of SFM, CGM, and DDGS at 15% in finisher diets of broilers with 21% and 20% CP levels is desirable for Cobb 500 strain from the growth performance aspects. Interestingly, incorporating a combination of SFM, CGM, and DDGS as APS into diets required less SBM supplementation and could be used for sustainable broiler production; moreover, it improves the economics of broiler production. However, further studies with different protein sources and higher levels of inclusion are needed to promote the use of the mixture of APS including CGM, SFM, and DDGS in broiler diets.
